# 1-(1*H*-Benzimidazol-2-yl)-4-nitro­benzene dimethyl­formamide solvate

**DOI:** 10.1107/S1600536809005315

**Published:** 2009-02-21

**Authors:** De-Hong Wu

**Affiliations:** a Ordered Matter Science Research Center, College of Chemistry and Chemical Engineering, Southeast University, Nanjing 210096, People’s Republic of China

## Abstract

In the title compound, C_13_H_9_N_3_O_2_·C_3_H_7_NO, the benzimidazole ring system and the benzene ring are essentially coplanar, forming a dihedral angle of 0.86 (5)°. The crystal packing is stabilized by an inter­molecular N—H⋯O hydrogen bond and a π–π stacking inter­action with a centroid–centroid separation of 3.685 (4) Å.

## Related literature

For general background to benzimidazole compounds, see: Zarrinmayeh *et al.* (1998 ▶); Gallagher *et al.* (2001 ▶); Howarth & Hanlon (2001 ▶).
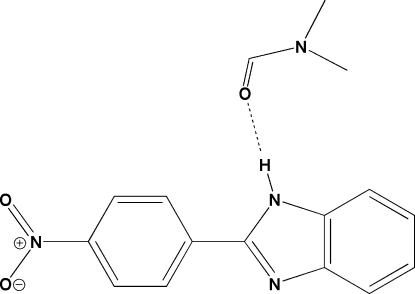


## Experimental

### 

#### Crystal data

C_13_H_9_N_3_O_2_·C_3_H_7_NO
*M* *_r_* = 312.33
Triclinic, 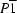

*a* = 6.6228 (13) Å
*b* = 10.601 (2) Å
*c* = 11.886 (2) Å
α = 84.534 (10)°
β = 74.13 (2)°
γ = 81.53 (3)°
*V* = 792.6 (3) Å^3^
*Z* = 2
Mo *K*α radiation
μ = 0.09 mm^−1^
*T* = 291 K
0.30 × 0.26 × 0.24 mm

#### Data collection

Rigaku Mercury2 diffractometer
Absorption correction: multi-scan (*CrystalClear*; Rigaku, 2005 ▶) *T*_min_ = 0.96, *T*_max_ = 0.98
7352 measured reflections
3102 independent reflections
1567 reflections with *I* > 2σ(*I*)
*R*_int_ = 0.045

#### Refinement

*R*[*F* ^2^ > 2σ(*F* ^2^)] = 0.060
*wR*(*F* ^2^) = 0.138
*S* = 1.01
3102 reflections
210 parameters
H-atom parameters constrained
Δρ_max_ = 0.12 e Å^−3^
Δρ_min_ = −0.20 e Å^−3^

### 

Data collection: *CrystalClear* (Rigaku, 2005 ▶); cell refinement: *CrystalClear*; data reduction: *CrystalClear*; program(s) used to solve structure: *SHELXS97* (Sheldrick, 2008 ▶); program(s) used to refine structure: *SHELXL97* (Sheldrick, 2008 ▶); molecular graphics: *SHELXTL* (Sheldrick, 2008 ▶); software used to prepare material for publication: *SHELXTL*.

## Supplementary Material

Crystal structure: contains datablocks I, global. DOI: 10.1107/S1600536809005315/rz2294sup1.cif

Structure factors: contains datablocks I. DOI: 10.1107/S1600536809005315/rz2294Isup2.hkl

Additional supplementary materials:  crystallographic information; 3D view; checkCIF report

## Figures and Tables

**Table 1:** Hydrogen-bond geometry (Å, °)

*D*—H⋯*A*	*D*—H	H⋯*A*	*D*⋯*A*	*D*—H⋯*A*
N1—H1*A*⋯O3^i^	0.90	1.89	2.753 (2)	161

Symmetry code: (i) 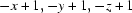.

**Fig. 1.**
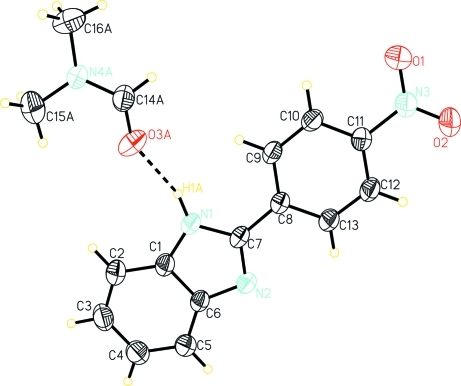
The molecular structure of the title compound, showing the atomic numbering scheme and 30% probability displacement ellipsoids. The A suffix for atoms O3, N4, C14, C15 and C16 denotes a transformation of (1 - x, 1 - y, 1 - z). The intermolecular N—H···O hydrogen bond is shown as a dashed line.

## supplementary crystallographic information

### Comment

Benzimidazole systems continue to attract much attention in chemical synthesis, structural science and applied medicinal research (Zarrinmayeh, *et al.*,1998; Gallagher *et al.*, 2001; Howarth & Hanlon, 2001). Here we report the crystal structure of the title compound, 1-(2-benzimidazolyl)-4-nitrobenzene dimethylformamide solvate.

The structural analysis shows that in the title compound (Fig. 1) the benzimidazole ring system and the benzene ring are essentially coplanar forming a dihedral angle of 0.86 (5)°. In the imidazole ring, the C7–N2 bond length of 1.327 (2) Å conforms to the value expected for a double bond. The dimethylformamide molecule bridges the benzimidazole ring system, forming an intermolecular N—H···O hydrogen bond (Table 1). The crystal packing is stabilized by aromatic π–π stacking interactions: Cp1···Cp2^i^ = 3.865 (4) Å; perpendicular interplanar distance: 3.374 (3) Å; Cp1···Cp2^i^ offset: 1.481 (3) Å (Cp1 and Cp2 are the centroids of the C1—C7 and C8—C13 aromatic rings, respectively; symmetry code: (i) -1+x, y, z).

### Experimental

The title compound was synthesized by refluxing 4-nitrobenzaldehyde (6.04 g, 4 mmol) and benzene-1,2-diamine (0.43 g, 4 mmol) in 40 ml absolute methanol for 10 h. After cooling to ambient temperature, the yellow solid formed was isolated and dried under vacuum (7.2 g, yield 75%). Single crystals suitable for X-ray structure analysis were obtained by slow evaporation of a dimethylformamide solution in air.

### Refinement

H atoms were placed in calculated positions (N—H = 0.86 Å; C—H = 0.93-0.96 Å), and refined using a riding model approximation with *U*_iso_ = 1.2 *U*_eq_(C, N) or 1.5 *U*_eq_(C) for methyl H atoms.

### Crystal data

**Table:**

C_13_H_9_N_3_O_2_·C_3_H_7_NO	*Z* = 2
*M**_r_* = 312.33	*F*(000) = 328
Triclinic, *P*1	*D*_x_ = 1.309 Mg m^−^^3^
Hall symbol: -P 1	Mo *K*α radiation, λ = 0.71073 Å
*a* = 6.6228 (13) Å	Cell parameters from 5280 reflections
*b* = 10.601 (2) Å	θ = 3.2–27.4°
*c* = 11.886 (2) Å	µ = 0.09 mm^−^^1^
α = 84.534 (10)°	*T* = 291 K
β = 74.13 (2)°	Block, yellow
γ = 81.53 (3)°	0.30 × 0.26 × 0.24 mm
*V* = 792.6 (3) Å^3^	

### Data collection

**Table:**

Rigaku Mercury2 diffractometer	3102 independent reflections
Radiation source: fine-focus sealed tube	1567 reflections with *I* > 2σ(*I*)
graphite	*R*_int_ = 0.045
Detector resolution: 13.6612 pixels mm^-1^	θ_max_ = 26.0°, θ_min_ = 3.2°
CCD_Profile_fitting scans	*h* = −8→8
Absorption correction: multi-scan (*CrystalClear*; Rigaku, 2005)	*k* = −13→12
*T*_min_ = 0.96, *T*_max_ = 0.98	*l* = −14→14
7352 measured reflections	

### Refinement

**Table:**

Refinement on *F*^2^	Primary atom site location: structure-invariant direct methods
Least-squares matrix: full	Secondary atom site location: difference Fourier map
*R*[*F*^2^ > 2σ(*F*^2^)] = 0.060	Hydrogen site location: inferred from neighbouring sites
*wR*(*F*^2^) = 0.138	H-atom parameters constrained
*S* = 1.01	*w* = 1/[σ^2^(*F*_o_^2^) + (0.0586*P*)^2^] where *P* = (*F*_o_^2^ + 2*F*_c_^2^)/3
3102 reflections	(Δ/σ)_max_ < 0.001
210 parameters	Δρ_max_ = 0.12 e Å^−^^3^
0 restraints	Δρ_min_ = −0.19 e Å^−^^3^

### Special details

**Table:**

Geometry. All e.s.d.'s (except the e.s.d. in the dihedral angle between two l.s. planes) are estimated using the full covariance matrix. The cell e.s.d.'s are taken into account individually in the estimation of e.s.d.'s in distances, angles and torsion angles; correlations between e.s.d.'s in cell parameters are only used when they are defined by crystal symmetry. An approximate (isotropic) treatment of cell e.s.d.'s is used for estimating e.s.d.'s involving l.s. planes.
Refinement. Refinement of *F*^2^ against ALL reflections. The weighted *R*-factor *wR* and goodness of fit *S* are based on *F*^2^, conventional *R*-factors *R* are based on *F*, with *F* set to zero for negative *F*^2^. The threshold expression of *F*^2^ > σ(*F*^2^) is used only for calculating *R*-factors(gt) *etc*. and is not relevant to the choice of reflections for refinement. *R*-factors based on *F*^2^ are statistically about twice as large as those based on *F*, and *R*- factors based on ALL data will be even larger.

### Fractional atomic coordinates and isotropic or equivalent isotropic displacement parameters (Å^2^)

**Table:**

	*x*	*y*	*z*	*U*_iso_*/*U*_eq_	
C1	−0.0895 (4)	0.3267 (2)	0.7884 (2)	0.0599 (6)	
C2	−0.2701 (4)	0.3902 (2)	0.8605 (2)	0.0798 (8)	
H2A	−0.2737	0.4732	0.8809	0.096*	
C3	−0.4438 (5)	0.3249 (3)	0.9006 (2)	0.0857 (8)	
H3A	−0.5668	0.3643	0.9497	0.103*	
C4	−0.4385 (4)	0.2011 (2)	0.8689 (2)	0.0798 (8)	
H4A	−0.5584	0.1597	0.8973	0.096*	
C5	−0.2605 (4)	0.1383 (2)	0.7965 (2)	0.0658 (7)	
H5A	−0.2587	0.0557	0.7756	0.079*	
C6	−0.0832 (4)	0.20250 (19)	0.75577 (18)	0.0540 (6)	
C7	0.2234 (4)	0.26232 (18)	0.67137 (18)	0.0535 (6)	
C8	0.4421 (4)	0.26378 (18)	0.60121 (18)	0.0520 (6)	
C9	0.5535 (4)	0.36846 (19)	0.5917 (2)	0.0605 (6)	
H9A	0.4864	0.4414	0.6308	0.073*	
C10	0.7583 (4)	0.3659 (2)	0.5264 (2)	0.0610 (6)	
H10A	0.8299	0.4365	0.5202	0.073*	
C11	0.8578 (4)	0.2568 (2)	0.46957 (19)	0.0564 (6)	
C12	0.7539 (4)	0.1508 (2)	0.4773 (2)	0.0667 (7)	
H12A	0.8226	0.0777	0.4389	0.080*	
C13	0.5471 (4)	0.1560 (2)	0.5427 (2)	0.0670 (7)	
H13A	0.4757	0.0855	0.5480	0.080*	
C14	0.7196 (4)	0.3189 (2)	0.2083 (2)	0.0751 (7)	
H14A	0.5807	0.3254	0.2545	0.090*	
C15	1.0088 (5)	0.1989 (3)	0.0766 (3)	0.1051 (10)	
H15A	1.0684	0.2774	0.0694	0.158*	
H15B	1.0053	0.1771	0.0006	0.158*	
H15C	1.0940	0.1320	0.1093	0.158*	
C16	0.6700 (5)	0.1095 (2)	0.1649 (3)	0.1028 (10)	
H16A	0.5303	0.1337	0.2136	0.154*	
H16B	0.7355	0.0352	0.2002	0.154*	
H16C	0.6612	0.0908	0.0891	0.154*	
N1	0.1073 (3)	0.36303 (15)	0.73324 (16)	0.0621 (5)	
H1A	0.1515	0.4383	0.7376	0.074*	
N2	0.1147 (3)	0.16428 (15)	0.68163 (16)	0.0564 (5)	
N3	1.0776 (3)	0.2533 (2)	0.39914 (18)	0.0720 (6)	
N4	0.7958 (3)	0.21395 (17)	0.15256 (17)	0.0647 (6)	
O1	1.1681 (3)	0.34815 (17)	0.39184 (17)	0.0937 (6)	
O2	1.1653 (3)	0.15534 (18)	0.35180 (19)	0.1073 (7)	
O3	0.8186 (3)	0.40844 (16)	0.20342 (19)	0.1109 (8)	

### Atomic displacement parameters (Å^2^)

**Table:**

	*U*^11^	*U*^22^	*U*^33^	*U*^12^	*U*^13^	*U*^23^
C1	0.0662 (17)	0.0574 (14)	0.0553 (15)	−0.0032 (13)	−0.0130 (13)	−0.0147 (12)
C2	0.086 (2)	0.0740 (17)	0.0737 (18)	−0.0067 (16)	−0.0048 (16)	−0.0276 (14)
C3	0.071 (2)	0.100 (2)	0.0763 (19)	−0.0065 (17)	0.0020 (15)	−0.0251 (16)
C4	0.076 (2)	0.0857 (19)	0.0749 (19)	−0.0169 (15)	−0.0098 (16)	−0.0081 (15)
C5	0.0683 (18)	0.0643 (15)	0.0641 (16)	−0.0103 (14)	−0.0141 (14)	−0.0081 (13)
C6	0.0637 (16)	0.0493 (12)	0.0487 (14)	−0.0006 (11)	−0.0159 (13)	−0.0085 (10)
C7	0.0644 (17)	0.0431 (12)	0.0561 (15)	0.0000 (11)	−0.0212 (13)	−0.0119 (11)
C8	0.0579 (15)	0.0467 (12)	0.0517 (14)	−0.0007 (11)	−0.0159 (12)	−0.0096 (10)
C9	0.0636 (17)	0.0448 (13)	0.0702 (16)	0.0021 (11)	−0.0139 (14)	−0.0144 (11)
C10	0.0647 (17)	0.0475 (12)	0.0715 (16)	−0.0054 (11)	−0.0179 (14)	−0.0103 (12)
C11	0.0571 (15)	0.0576 (14)	0.0540 (14)	−0.0015 (12)	−0.0149 (12)	−0.0094 (11)
C12	0.0693 (18)	0.0543 (14)	0.0756 (18)	−0.0022 (13)	−0.0139 (15)	−0.0237 (12)
C13	0.0667 (18)	0.0564 (14)	0.0773 (17)	−0.0107 (12)	−0.0098 (15)	−0.0243 (13)
C14	0.0786 (19)	0.0670 (16)	0.0733 (18)	0.0010 (15)	−0.0107 (15)	−0.0157 (14)
C15	0.086 (2)	0.116 (2)	0.097 (2)	0.0080 (18)	−0.0026 (19)	−0.0213 (18)
C16	0.126 (3)	0.0753 (18)	0.118 (3)	−0.0349 (19)	−0.041 (2)	−0.0015 (17)
N1	0.0643 (13)	0.0478 (10)	0.0726 (13)	−0.0072 (9)	−0.0103 (11)	−0.0195 (9)
N2	0.0595 (13)	0.0482 (10)	0.0629 (13)	−0.0041 (9)	−0.0176 (11)	−0.0098 (9)
N3	0.0658 (15)	0.0694 (14)	0.0772 (15)	−0.0028 (12)	−0.0122 (12)	−0.0158 (12)
N4	0.0693 (14)	0.0548 (11)	0.0672 (13)	−0.0039 (10)	−0.0117 (11)	−0.0145 (10)
O1	0.0768 (13)	0.0837 (12)	0.1160 (16)	−0.0240 (11)	−0.0070 (11)	−0.0164 (11)
O2	0.0802 (14)	0.0926 (14)	0.1324 (18)	−0.0057 (11)	0.0111 (12)	−0.0483 (13)
O3	0.1330 (19)	0.0708 (12)	0.1336 (18)	−0.0294 (12)	−0.0241 (14)	−0.0360 (12)

### Geometric parameters (Å, °)

**Table:**

C1—N1	1.381 (3)	C10—H10A	0.9300
C1—C2	1.389 (3)	C11—C12	1.385 (3)
C1—C6	1.400 (3)	C11—N3	1.464 (3)
C2—C3	1.379 (3)	C12—C13	1.374 (3)
C2—H2A	0.9300	C12—H12A	0.9300
C3—C4	1.393 (3)	C13—H13A	0.9300
C3—H3A	0.9300	C14—O3	1.219 (3)
C4—C5	1.377 (3)	C14—N4	1.313 (3)
C4—H4A	0.9300	C14—H14A	0.9300
C5—C6	1.392 (3)	C15—N4	1.448 (3)
C5—H5A	0.9300	C15—H15A	0.9600
C6—N2	1.392 (3)	C15—H15B	0.9600
C7—N2	1.327 (2)	C15—H15C	0.9600
C7—N1	1.368 (2)	C16—N4	1.454 (3)
C7—C8	1.462 (3)	C16—H16A	0.9600
C8—C13	1.387 (3)	C16—H16B	0.9600
C8—C9	1.400 (3)	C16—H16C	0.9600
C9—C10	1.364 (3)	N1—H1A	0.8998
C9—H9A	0.9300	N3—O2	1.220 (2)
C10—C11	1.381 (3)	N3—O1	1.229 (2)
			
N1—C1—C2	132.3 (2)	C12—C11—N3	119.2 (2)
N1—C1—C6	105.77 (19)	C13—C12—C11	118.5 (2)
C2—C1—C6	121.9 (2)	C13—C12—H12A	120.7
C3—C2—C1	117.2 (2)	C11—C12—H12A	120.7
C3—C2—H2A	121.4	C12—C13—C8	121.7 (2)
C1—C2—H2A	121.4	C12—C13—H13A	119.2
C2—C3—C4	121.3 (2)	C8—C13—H13A	119.2
C2—C3—H3A	119.4	O3—C14—N4	124.4 (3)
C4—C3—H3A	119.4	O3—C14—H14A	117.8
C5—C4—C3	121.8 (3)	N4—C14—H14A	117.8
C5—C4—H4A	119.1	N4—C15—H15A	109.5
C3—C4—H4A	119.1	N4—C15—H15B	109.5
C4—C5—C6	117.8 (2)	H15A—C15—H15B	109.5
C4—C5—H5A	121.1	N4—C15—H15C	109.5
C6—C5—H5A	121.1	H15A—C15—H15C	109.5
C5—C6—N2	130.4 (2)	H15B—C15—H15C	109.5
C5—C6—C1	120.1 (2)	N4—C16—H16A	109.5
N2—C6—C1	109.5 (2)	N4—C16—H16B	109.5
N2—C7—N1	112.44 (19)	H16A—C16—H16B	109.5
N2—C7—C8	124.12 (18)	N4—C16—H16C	109.5
N1—C7—C8	123.44 (19)	H16A—C16—H16C	109.5
C13—C8—C9	117.9 (2)	H16B—C16—H16C	109.5
C13—C8—C7	119.0 (2)	C7—N1—C1	107.00 (17)
C9—C8—C7	123.09 (19)	C7—N1—H1A	126.3
C10—C9—C8	121.5 (2)	C1—N1—H1A	126.7
C10—C9—H9A	119.3	C7—N2—C6	105.30 (17)
C8—C9—H9A	119.3	O2—N3—O1	122.4 (2)
C9—C10—C11	119.0 (2)	O2—N3—C11	118.7 (2)
C9—C10—H10A	120.5	O1—N3—C11	118.9 (2)
C11—C10—H10A	120.5	C14—N4—C15	120.7 (2)
C10—C11—C12	121.5 (2)	C14—N4—C16	121.4 (2)
C10—C11—N3	119.3 (2)	C15—N4—C16	117.9 (2)

### Hydrogen-bond geometry (Å, °)

**Table:**

*D*—H···*A*	*D*—H	H···*A*	*D*···*A*	*D*—H···*A*
N1—H1A···O3^i^	0.90	1.89	2.753 (2)	161

Symmetry codes: (i) −*x*+1, −*y*+1, −*z*+1.
